# 

**Published:** 1894-09-08

**Authors:** 


**Notices of Births, Deaths, and Marriages** are inserted in this column at a charge of 2s. 6d. for an announcement not exceeding four lines.

**Notice to Advertisers and Subscribers.**

All Advertisements, Orders for Copies of The Hospital, and Business Communications should be addressed to **The Publisher, NOT TO THE EDITOR,** The Hospital, 428, Strand, W.C.

The Cost of Subscription, post free, is as follows:—Per week, 2½d.; per quarter, 2s. 8d.; per half-year, 5s. 3d.; per annum, 10s. 6d. Foreign:—Per Annum, 12s. 8d.; payable, in all cases, in advance.

**NOTICE TO CORRESPONDENTS.**

All MS., Letters, Books for Review, and other matters intended for the Editor should be addressed The Editor, The Lodge, Porchester Square, London, W.

The Editor cannot undertake to return rejected MS., even when accompanied by stamped directed envelope.

**"Burdett's Hospital Annual," 1894.**

Fifth year of publication. 900 pp. Boards, gilt lettering. A most complete guide to British, Colonial, Indian, and American Hospitals, Dispensaries, Nursing and Convalescent Institutions, and Asylums. Price 5s. Published by The Scientific Press (Limited), 428, Strand, W.C.

**NOTICE.—The Index for Vol. XV. (ending March 31st, 1894) is on sale at the Office of this Journal, price 2½d., post free.**

### Scraps and Gleanings.

A most successful garden fête was held lately in the grounds of Poltimore Park, near Exeter, in aid of the Rural District Nursing Fund.

A five days' flower show and military tournament has been held at Willesden Green in aid of the maintenance fund of the Willesden Cottage Hospital.

The profits of the charity concert given by Madame Patti, at Swansea, were £529. Of this the poor of Craig-yr-Nos receive £176, and the Swansea Hospital £353.

The cabmen of Exeter have made a special effort this year on behalf of the Hospital Saturday fund. The St. Thomas' cabmen collected £5 by means of a box at their shelter.

Preparations are being made at Birmingham for a suitable reception for the Duke and Duchess of York on the occasion of the laying of the foundation stone of the New General Hospital.

At the annual meeting of the Cornwall Convalescent Home for Men at Perranporth, held this month at Truro, the statement of accounts was most satisfactory, the credit balance being £174.

It is proposed to build a workhouse hospital for Halifax at Skircoat which will accommodate 400 patients. It is suggested to utilise the present hospital for much needed workhouse accommodation.

The Local Government Board have absolutely refused to sanction the proposed loan for the purpose of establishing a joint isolation hospital for the districts of Richmond and of Heston and Isleworth.

The foundation stone has been laid for a cottage hospital at Wood Green in a pleasant spot on the New Southgate side of Bounds' Green Road. The cost of the building, which is £2,000, has been defrayed by Mr. Passmore Edwards.

In recognition of the gift of £1,000 to the Meath Hospital by the late Mr. McComar, the committee have arranged to dedicate a bed in one of the wards to his memory, over which is to be placed a brass tablet engraved with his name.

Upwards of £115 was collected this year at Llandudno on Hospital Saturday in aid of the Sarah Nicol Memorial Cottage Hospital. A contribution of £100 has been sent by Mrs. Williams Jones-Barry to form a nucleus for an endowment fund.

The State of New York has recently passed a law authorising the use of Dr. Koch's tuberculin in a novel fashion. Two or three drops injected beneath the skin of a cow will decide in a few hours whether there is danger of tuberculous infection in the milk.

The Stockton Hospital Committee have examined the list of workmen not subscribing to the hospital, and issued circulars inviting the non-subscribers to do so. The result of the collections on Hospital Sunday was £102 16s. 4d., being £4 less than last year.

A Convalescent Children's Hospital at Broadstairs is being erected by the well-known torpedo-boat builder, Mr. Yarrow. It is said that the hospital will cost about £120,000. The plans are furnished and the details superintended gratis by Mr. Barrow Emanuel.

In the annual report of the Cottage Hospital at Macclesfield, the Chairman referred to the satisfactory work and position of the institution. A sum of £50 had been transferred to the reserve fund from the balance of last year's account. The reserve fund now stands at £549.

Plans are already prepared for the proposed new general hospital for Kettering. The hospital will be commenced as soon as the subscriptions, which are mounting up well, are augmented by another £250. At present the nearest hospital is fourteen miles from Kettering and the surrounding villages.

Arrangements are being made for a new infirmary for co. Tyrone. Upwards of £2,000 has already been subscribed, and it is suggested to collect from the whole county to make up the remaining £4,000 still required for the building fund. Hospital collections are to take place in October.

Large crowds gathered at the Heights, at Dover, on Hospital Sunday to witness a drumhead service held in aid of the Dover Hospital. There was a procession of Friendly Societies in the afternoon which marched to a service held above Godwyne Road. The collections amounted to £9 10s. 7d.

The Middlesex Hospital has just held the quarterly general court of the governors. It was stated that a petition was to be presented to the Corporation of the City of London for a grant in aid of the hospital funds. The convalescent home at Colchester, now in progress, is to cost £18,475.

The annual bazaar in aid of the Convalescent Home for Men at Llanfairfechan was a considerable financial success this year. In the evening of the last day a grand concert was held. This year's bazaar was chiefly held with a view to assist in clearing off the mortgage of £1,000 on the Home property.

An industrial exhibition is to be held in the Drill Hall at Sunderland in November, which will include a collection of marine, electrical, and engineering exhibits. The local charities generally are to benefit by the proceeds, but the Sunderland Infirmary, which celebrates its centenary this year, will have a special interest in the exhibition.

A hospital parade took place at Barnstaple on the last Saturday in August. A novel entertainment was the inclusion of industrial tableaux representing the manufacture of the celebrated Barum ware and other commodities. The Ambulance Corps contributed to the proceedings by a demonstration of first aid to the injured. The receipts were most gratifying.

An inquiry has recently been held relative to the establishment of a joint isolation hospital for the following districts: The Bromsgrove Rural Sanitary Authority, the Bromsgrove County Local Board, the Bromsgrove Town and Redditch Local Government Districts, the Droitwich Town Council, and the Droitwich Rural Sanitary Authority. The cost of the proposed hospital is estimated at about £8,000.

At the quarterly meeting of the Belfast Royal Infirmary at the end of last month, the receipts from all sources were shown to be £9,523 17s. 11d. Of this £1,000 had been set apart for external extension, and £1,000 invested. It was stated that there was a substantial increase in subscriptions from the working classes. The debt for the eleven months ending July 30th amounts to £722 3s. 3d., which includes the deficit of the previous year.

### THE STUDENT OF MEDICINE.

### APPOINTMENTS.

P. S. Abraham, M.D.Dub., B.Sc.Lond., F.R.C.S.I., appointed Dermatologist to the West London Hospital. J. H. Allard, L.R.C.P.Edin., M.R.C.S.Eng., appointed Medical Officer of Health to the Tewkesbury Urban Sanitary District. W. M. Anderson, M.B., appointed Medical Officer of the Infirmary of the Parish of Chelsea. G. M. Arkle, L.S.A.Lond., appointed Medical Officer for the Walton Workhouse of the West Derby Union. H. Ashton, L.R.C.P., L.R.C.S.Edin., appointed Medical Officer for the Fourth District of the Oldham Union. S. W. Brook, M.B., B.Ch.Vict., appointed House Surgeon to the Manchester Royal Infirmary. R. Cattley, M.B.Durh., D.P.H. Camb., appointed Medical Officer of Health to the York City Council. W. W. Clemesha, M.B., B.Ch.Vict., appointed House Surgeon to the Manchester Royal Infirmary. Mr. J. E. Couison, appointed Second Assistant Medical Officer to the Workhouse and Infirmary of the St. George's Union. G. B. Griffiths, L.R.C.P.Lond., M.R.C.S.Eng., appointed Resident Medical Officer to the Eastern Dispensary, Bath. H. O. Hughes, L.R.C.P., L.R.C.S.Edin., appointed Medical Officer for the Abergele District of the St. Aspah Union.

### VACANCIES.

#### Resident Medical Officers.

City of London Hospital for Diseases of the Chest, Victoria Park, E.—House Physician. Board, residence, and allowance for washing provided. Appointment for six months. Applications to the Secretary, at the Office, 24, Finsbury Circus, E.C., by September 13th.

North Wales Asylum, Denbigh.—Second Assistant Medical Officer. Salary £100 per annum, with board, washing, and apartments. Applications to W. Bark, Clerk to the Visitors, by September 12th.

Royal Alexandra Hospital for Sick Children, Brighton.—House Surgeon; doubly qualified. Salary £80 per annum, with board, lodging, and washing, but no stimulants. Applications to the Chairman of the Medical Committe by September 8th.

#### Non-Resident Medical Officers.

St. Bartholomew's Hospital.—Ophthalmic Surgeon, must be F.R.C.S. Eng. Applications and testimonials to W. Cross, Clerk, by September 10th.

### UNIVERSITIES AND COLLEGES.

#### Royal University of Ireland.

FACULTY OF MEDICINE: SUMMER, 1894.—The following candidates have been adjudged to have passed the First Examination in Medicine: J. F. Begley, University College, Dublin; N. J. Blaney, University College, Dublin; D. Brown, Queen's College, Belfast; J. W. Brown, Queen's College, Belfast; F. C. Bullen, Queen's College, Cork; P. J. Burke, University College, Dublin; P. Canning, B.A., University College, Dublin; J. J. S. Carbery, University College, Dublin; W. V. Coppinger, School of Physic, Trinity College, Dublin; J. J. Crowley, B.A., Queen's College, Cork; J. Daly, Queen's College, Cork; R. A. L. Graham, Queen's College, Belfast; A. J. Hewitt, Queen's College, Galway; G. Jefferson, Queen's College, Belfast; R. Kerr, Queen's College, Belfast; A. E. Knight, Queen's College, Belfast; M. J. M'Donough, University College, Dublin; K. M'Gahey, Queen's College, Belfast; A. B. M'Master, Queen's College, Belfast; J. W. D. Megaw, Queen's College, Belfast; D. Murphy, Queen's College, Cork; W. O'S. Murphy, Queen's College, Cork; J. Murray, University College, Dublin; E. O'Callaghan, University College, Dublin; W. Paisley, Queen's College, Galway; O. M.

THE STUDENT OF MEDICINE—*continued.*

Praeger, Queen's College, Belfast; F. S. Scott, Queen's College, Galway; A. M. Staunton, Queen's College, Galway; R. Steen, Queen's College, Belfast; Isabel A. Tate, Queen's College, Belfast; J. Walsh, Queen's College, Cork; S. H. E. Walshe, Queen's College, Belfast; K. H. White, Queen's College, Cork; and R. Whyte, Queen's College, Belfast.

Honours.

Botany.—First Class: P. Canning, B.A., University College, Dublin; J. Murray, University College, Dublin; and J. F. Begley, University College, Dublin. Second Class: N. J. Blaney, University College, Dublin; and W. V. Coppinger, School of Physic, Trinity College, Dublin.

Zoology.—First Class: N. J. Blaney, University College, Dublin; and P. Canning, B.A., University College, Dublin. Second Class: J. F. Begley, University College, Dublin; and J. Murray, University College, Dublin.

Chemistry.—First Class: P. Canning, B.A., University College, Dublin; and N. J. Blaney, University College, Dublin. Second Class: J. F. Begley, University College, Dublin; J. W. Brown, Queen's College, Belfast; P. J. Burke, University College, Dublin; and J. Murray, University College, Dublin.

Experimental Physics.—First Class: N. J. Blaney, University College, Dublin; J. W. D. Megaw, Queen's College, Belfast; P. Canning, B.A., University College, Dublin; J. W. Brown, Queen's College, Belfast; and J. F. Begley, University College, Dublin. Second Class: W. V. Coppinger, School of Physic, Trinity College, Dublin; and J. Murray, University College, Dublin.

Exhibitions (Candidates who upon their answering were qualified for exhibitions, the names of those disqualified by standing or otherwise are printed in italics).—First Class, £20; *P. Canning, B.A., University College, Dublin*; and N. J. Blaney, University College, Dublin. Second Class, £10 each: J. F. Begley, University College, Dublin.

